# Optimizing the social utility of judicial punishment: An evolutionary biology and neuroscience perspective

**DOI:** 10.3389/fnhum.2022.967090

**Published:** 2022-09-12

**Authors:** Daniel A. Levy

**Affiliations:** Baruch Ivcher School of Psychology, Reichman University – IDC, Herzliya, Israel

**Keywords:** punishment, evolution, society and culture, ethology and behavioral ecology, justice

## Abstract

Punishment as a response to impairment of individual or group welfare may be found not only among humans but also among a wide range of social animals. In some cases, acts of punishment serve to increase social cooperation among conspecifics. Such phenomena motivate the search for the biological foundations of punishment among humans. Of special interest are cases of pro-social punishment of individuals harming others. Behavioral studies have shown that in economic games people punish exploiters even at a cost to their own welfare. Additionally, neuroimaging studies have reported activity during the planning of such punishment in brain areas involved in the anticipation of reward. Such findings hint that there is an evolutionarily honed basic drive to punish social offenders. I argue that the transfer of punishment authority from the individual to the group requires that social offenders be punished as a public good, even if such punishment is not effective as retribution or deterrent. Furthermore, the social need for punishment of offenders has implications for alternatives to incarceration, publicity of punishment, and judicial structure.

## Introduction

*Homo juridicus* – the law-governed person – lives by the belief that their decisions and opinions are rooted in a system of values and rational considerations. In recent years, however, trends in the social sciences and life sciences have challenged this belief. Sociobiology and evolutionary psychology – disciplines that have developed relatively recently – claim that many human opinions ostensibly based on logic and judgment often express behavioral tendencies and feelings that stem from adaptive impulses shaped by evolution (Buckholtz and Marois, 2012). One way to identify the sources of our behaviors is through comparative psychology, including ethology, the study of animal behavior. If we identify behaviors common to humans and to other animals (that ostensibly do not operate based on a system of conscious rational considerations), then the motives for that behavior may be adaptive processes that serve survival and reproduction (Mobbs et al., 2018). Many examples of this phenomenon can be found in sexual behavior: all the phenomena that characterize the relationship between the sexes in humans (monogamy, polygamy, polyandry, jealousy, loyalty, courtship gifts, dressing for the purpose of attracting a partner, etc.) can be found in a wide variety of animals, and have a clear evolutionary explanation (Rubenstein and Alcock, 2019).

It is not only in the field of behaviors related to seemingly basic biological processes, such as reproduction, that evolutionary bases can be found. Such effects can also be found in societal institutions. This article deals with the subject of punishment. I chose to examine the institution of punishment because it is an important component of the legal system in every society and culture, and should ostensibly reflect values and rules of cooperative action that are especially important to any society. Elsewhere I have dealt with the argument that people’s approach to punishment reflects their beliefs regarding free will (Levy, 2003). As we shall see, though, social punitive behavior also exists in other animals. Furthermore, recent studies have identified some of the neural infrastructures of punitive behavior in humans, and these findings suggest that conscious rational considerations are only the tip of the iceberg of this behavior.

As the following portions of the article will espouse some unconventional and seemingly less-progressive approaches to instruments of societal and legal punishment, it is important at the outset to note that legal punishments often sadly result from the failures of societies to be just and charitable to all their members. Violent and exploitative crimes are likely to occur in environments in which individuals are oppressed, frustrated, and deprived of the ability to thrive by the dint of their own efforts. The optimal state of society is achieved by affordance of truly equal access to resources, institutional benevolence toward those less privileged because of innate and environmental factors, and proactive steps to address historical sources of inequality. Those values-laden but eminently effective approaches to public policy should be our first line of action in creating societies in which all participants are safe and at peace. Punishment, and the societal institutions that employ it, should be a last resort. Given this strong reservation, it remains to be determined how judicial punishment can be most effective when it is unavoidable.

## Legal-philosophical approaches to punishment

Before examining the biological basis of punishment, it will be helpful to briefly review the principles of punishment in the two regnant legal-philosophical approaches. The *consequentialist* approach, based on the utilitarian tradition of Jeremy Bentham and his school (Bentham, 1789/1970), emphasizes deterrence, removing dangerous people from society, and obliging offenders to undergo rehabilitation. In contrast, the *retributive* approach, identified with the teachings of Immanuel Kant (1797/1991), holds that criminals should be punished for the evil deeds they have done, in accordance with the “inner evil” they reflect, regardless of the consequences of punishment on the future situation (Bedau, 2008)^1^.

### The consequentialist approach

According to utilitarian philosophy, the purpose of all laws is to maximize the aggregate welfare of society. As any act of punishment creates suffering (and thus reduces aggregate welfare), it will be justified only if it leads to the prevention of greater suffering. In contrast to the retributive approach, which focuses on the characteristics of the offender’s past actions, the consequential approach focuses on the future benefits that can be derived from the act of punishment – to the offender, the victim, and society as a whole (Hudson, 2003).

The consequentialist approach requires punishment to satisfy a range of social goals (Haist, 2008): (1) Deterrence – punishment is just if it leads to a decrease in future crime, and deters potential offenders. (2) Crime prevention – removing offenders from open society for a certain period of time, to deny them the ability to cause further damage. The punishment imposed on the offender stems in part from assessment of future offenses they may commit. This goal is achieved in contemporary cultures mainly through imprisonment^2^. (3) Rehabilitation – this approach focuses on correction, and aims to change the offender’s attitude toward crime. It does not content itself with removing the offender from society, but tries to reduce the chance that they will return to engaging in criminal activity, by providing individual or group treatment. In addition, according to this approach, uniform punishment should be avoided as much as possible, and efforts should be made to adapt the form and severity of the punishment to the particular offender.

### The retributive approach

In contrast to the consequentialist approach, this approach focuses on the past, and does not address future consequences of punishment, including deterrence or rehabilitation of the offender. The retributive approach has two aspects: one, understanding retribution as a kind of societal revenge^3^ ; the other, retribution meaning an appropriate ratio between the severity of the act and the degree of punishment (Hudson, 2003). Viewing retribution as societal revenge, there is not necessarily a match between the severity of the act and the degree of punishment, and revenge is done entirely to satisfy the punisher. When it comes to retribution as “cosmically” appropriate treatment, there should be a match between the severity of the act and the degree of punishment. Punishment will not necessarily satisfy the punisher’s desire for revenge – rather, some objective moral boundary is set for the punitive acts. The rule of “An eye for an eye,” expressed as early as biblical times (Exodus 21:24), expresses, among other things, the principle of act-appropriate retaliation, hence its Roman name *Lex Talionis*. The underlying idea is a balance between crime and punishment - measure for measure.

The retributive approach has been supported by philosophical models, and its prominent proponents are Kant (e.g., Kant, 1797/1991) and Hegel (e.g., Hegel, 1832/1988). Kant’s ethics is based on the “categorical imperative,” which requires every person: “Act only according to the same practical rule which, by accepting it, you will also want, because it will be a general law.” That is, humans must act in the same way they would want other humans to act, as a universal norm. Hence, the justification for punishment is that the offender deserves punishment, since s/he has transgressed his or her own moral standards. Kant teaches that punishment should never be imposed as a means of promoting any benefit to society or to the offender, since humans are important in themselves: “Juridical punishment can never be administered merely as a means of promoting another good.” (Kant, 1797/1991). This theory seems to be a complete antithesis to the consequentialist approach. It completely ignores the consequences of punishment and its deterrent component, and is based entirely on considering the act of transgression and the appropriate punishment for it (Moore, 2010).

According to Hegel, the revenge component is irrelevant to punishment. Punishment is seen as the *right of the punished*, and constitutes a means of recognizing their humanity and responsibility toward society. The punishment is given out of respect for the punished, who has chosen to infringe on the mutual responsibility between the individuals in society. Punishment is intended to balance some cosmic moral equilibrium, which was damaged when the offense was committed and the positive element of justice was denied. Like Kant, Hegel believed that punishment should be determined by past transgressions, and should refrain from addressing future benefits (Stillman, 1976).

Punishment based on the principle of retribution was manifested in the United States in the 1970s, under the policy of “just deserts.” This policy developed in the wake of findings that imprisonment did not achieve its intended corrective purpose, which led to demands that it be set aside. A committee addressing this issue proposed to give “just deserts” punishment, to be determined according to two components of the offense: the actual damage caused and the degree of guilt (von Hirsch, 1992).

Retaliatory punishment is expressed in various pieces of Israeli legislation and case law, both overtly and covertly, despite society’s perception of the goals of revenge and retribution as archaic and primitive. A clear example of retributive punishment is punitive damages. Ordinary compensation is intended to reimburse damages. In contrast, punitive damage fines focus on the behavior of the offender. Their purpose is “to punish the offender for his harmful behavior and thus to express aversion to it” (Englard et al., 1976; Kuklin, 1989). At present, there is no explicit determination in Israeli law regarding the court’s authority to impose punitive damages, although it seems that this trend is developing. Evidence of this can be found in Israel’s proposed Code of Civil Law (2004), which gives the court the power to award compensation – for example (Section 462): “The court may award the injured party compensation that does not depend on the degree of damage, if it finds that the violation was committed maliciously.”

An extreme example of legislation specifically expressing the retributive justification for punishment is the State of Israel’s Nazis and Nazi Collaborators Punishment Law (1950). This law explicitly endorses feelings of retaliation, and even of revenge, and it seems that the goals of deterrence, protection of the public and correction of the offender were secondary or even negligible factors in its enactment.

Commonly, an attempt is made to distinguish between revenge and proportional treatment in punishment. As indicated by the following rulings, while the appropriate ratio component is perceived as legitimate, the revenge component is perceived as archaic and outdated. The courts refrain from basing their decision on considerations of revenge, but accept considerations of “just deserts” as relevant justifications:

In the serious nature of the offense before us and the circumstances of its commission, we may not ignore the consideration of retribution, which expresses an appropriate relationship between the severity of the offense and the severity of the punishment, not as an act of revenge, but as an expression of revulsion and disgust regarding the embezzlement by the defendant of public funds that were given to him due to absolute confidence in his honesty and fairness (Anonymous v. State of Israel, 1981).

And also:

Indeed, retribution as revenge is not a legitimate aspect of judicial punishment; but when it comes to punishment for a serious crime, such as the one before us, retribution expressing abhorrence of and revulsion by the crime, which warps the basic nature of human society, is appropriate. The severity of the punishment expresses condemnation of the act and abhorrence of it (Fadida v. State of Israel, 1993).

How, though, can one distinguish between a punishment imposed out of “disgust” and “revulsion” and one imposed from revenge considerations? Despite the notion that punishment for retaliatory revenge is archaic and primitive, it seems that the Israeli legislature in many cases obliges a judge to hand down a retaliatory punishment, and the judges themselves, although they do not necessarily openly admit it, also prescribe punishment based on considerations of revenge and retribution.

### Identification of punitive motives

Theoretically, it can be argued that there need not exist any contradiction between the consequentialist and the retributive approaches, and that they can complement each other in guiding punitive decisions. However, Carlsmith et al. (2002) pointed out that different aspects of a crime would lead to different decisions in accordance with these two principles. Retributive considerations focus on the degree of damage to the victim and the intention of the criminal as the critical factors, while the consequentialist view would focus on public awareness of the crime and the chances of it being discovered^4^. Carlsmith et al. (2002) have shown that ordinary citizens often claim that what guides them in thinking about punishment are utilitarian (consequentialist) considerations, not retaliatory considerations (“doing justice”). In practice, though, when these considerations lead to different sentences – laypeople usually act out of retributive considerations, not considerations of prevention. What could be the reason for this cognitive-behavioral contradiction? It remains to be seen whether a biological understanding of the punishment mechanisms can explain the phenomenon.

## The ethology of punishment

Punishment in response to harm to the good of the individual or group exists not only among humans but also among other species of social animals. One may wonder: Is it appropriate to talk about “punishment” in monkeys, other mammals, birds or even insects? Many – but not all – biologists claim that it is indeed possible. One biological definition of punishment is the response of individuals or groups “to actions likely to lower their fitness with behavior that reduces the fitness of the instigator and discourages or prevents him or her from repeating the initial action” (Clutton-Brock and Parker, 1995)^5^. There is no reference to laws or legal systems in this definition, but it seemingly manages to describe very interesting behaviors in the world of animals, and as we shall see - punishment in humans as well. Below we will review some cases in which such behaviors have been documented.

### Punishment to prevent disruption of reproductive resources

Perhaps the most basically adaptive cases of animal punishment are found in the context of reproductive behaviors. The naked mole rat conducts a special form of reproduction in which one female in the group gives birth, and others care for her and her offspring. These maternal queens will attack workers who do not work hard enough for the benefit of the group (Reeve, 1992). Among Polistes wasps, the main hive queen attacks lazy workers by chasing, wrestling, pushing and biting them (Reeve and Gamboa, 1987). Among superb fairy wrens there is a class of helpers, who do not start families themselves, but help raise other chicks, usually their brothers and sisters, by collecting food. Helpers who were absent from the group during the season of caring for chicks suffered attacks from the dominant male when they were returned to their place, but not if they were absent while they were not expected to assist in feeding chicks (Mulder and Langmore, 1993). Aggression, especially by dominant animals, can therefore be seen as a way to maintain their reproductive status relative to other members of the group. However, punishment is not just the domain of dominant animals. Polistes wasps exhibit a hierarchy in which several laying queens participate. The main queen restricts laying by the subordinate queens by eating about a third of their eggs. If the main queen exceeds this amount in her eating, her sisters often attack her (Reeve and Nonacs, 1992).

Conservation of important resources for subsistence and reproduction is done not only by repelling exploiters, but also by post-facto retaliation. Birds take materials for building nests and food from each other. Some species, such as the cuckoo, lay their eggs parasitically in the nests of other birds. In response, long chases are sometimes conducted by the bird affected by these thefts and attempts at exploitation (Clutton-Brock and Parker, 1995). Mammal cubs living in large groups will sometimes try to supplement the lack of breastfeeding by taking advantage of another mother from the group. In the case of elephant seals, this attempt may provoke a violent and even murderous reaction on the part of the exploited mother (Reiter et al., 1978). Adult moose control a harem of females, preventing young males from mating with them. The dominant males will not only persecute younger males who try their luck, but will chase them for quite a distance, attack them with their antlers, and sometimes even kill them (Clutton-Brock et al., 1988). Clutton-Brock (2017) has recently argued that retaliatory aggression among non-human animals serves a similar adaptive deterrence function as vengeful behaviors do in the human context.

### Punishment to maintain social order and cooperative behaviors

Punitive actions of animals are intended in some cases to bring about an increase in cooperation between members of the same species (Raihani et al., 2012). For example, macaque monkeys live in groups, and individuals who discover preferred food sources tend to make special food calls to inform the rest of the group. Monkeys who do not share their good fortune with the rest of the group in by signaling have a higher probability of falling victim to group aggression than those who do signal (Hauser and Marler, 1993). Chimpanzees lead extremely complex social lives, including building coalitions to gain access to desirable resources. They may attack allies who do not support them in confrontations with competitors (de Waal, 1992). Vervet monkeys of the same group may punish each other in sex-specific manners during intergroup encounters, in response to initiation of attacks or non-participation in group defense (Arseneau-Robar et al., 2016, 2018).

In several human justice systems (contemporary as well as historical), family members of a criminal have paid a price for their relative’s crime^6^. Among vervet monkeys, adult females that have been deprived of food sources will seek out and attack the family members of their dispossessor (Cheney and Seyfarth, 1990). We can understand this in evolutionary terms, as reflecting struggle between members of gene pools for reproductive fitness – one of the foundations of biological thinking.

### Third-party interventions and group-norm enforcement

Additional complexities exist in apes, in the form of what might be called “third-party intervention.” In some cases, dominant males and females will interfere in fights between subordinates in their groups, possibly for the purpose of maintaining group order (Ehardt and Bernstein, 1992). Such “policing” behavior has been reported in chimpanzees, bonobos, captive orangutans, and additional species. Various theories have been proposed to explain the existence of such costly behavior [reviewed by von Rohr et al. (2012)]. These include maintenance of group stability by high-ranking individuals who have the most to gain at the lowest cost due to their power, in the form, e.g., of sexual benefits to a male enforcer resulting from the prevention of violence between females. Another motivation might be the assurance of dominance: dominance-ambitious males often support the winners, but currently dominant males support the losers in the fight (Jennings et al., 2009). Subordinate chimpanzees may draw the attention of dominant males to the behavior of other members of the group that act against them, as if to mobilize the forces of justice to establish order (De Waal, 1982).

An interesting case of punishment used to maintain the stability of an economic ecosystem is found among cleaner wrasses. These reef-dwelling fish offer a parasite-cleaning service to other species. A male will control a large territory incorporating ‘service stations’ of several female cleaners. If the wrasses exploit clients by eating valuable mucus rather than ectoparasites, those clients will retaliate against the cleaners, and not return to that locus. This creates a conflict, in that the female cleaners might risk the cost of exploitation to take advantage of the immediate preferred food, at a cost to the male of loss of that client. Males were found to punish females who did so, investing that effort to prevent the loss of those or other clients in the future (Raihani et al., 2010). In a sense this is third party punishment, in that the male is punishing the female who is exploiting the client, but the motivation of that punishment is to protect the resources of the male enforcer.

Ravens (birds known for their impressive cognitive abilities) also show evidence of third-party involvement in order to maintain social order. There is a social norm that any raven that holds food can keep it, regardless of the status of other ravens present (unlike other birds, which have a “pecking order”). A raven that violates this rule and attempts to take some of the food will be attacked by a third raven, who is not necessarily a relative of the victim. The punisher does not gain any direct personal benefit from punishing the thief. It seems, then, that maintaining social norms is so important to ravens that they will punish violation of the norm even if they themselves have not been harmed (Heinrich, 1999). Similarly, Fraser and Bugnyar (2012) observed ravens exhibiting agonistic support (defined as a third party attacking a conflict participant, thus supporting the other), which resulted in long-term (but not short-term) reciprocity. That behavior exemplifies a possibly more general biological basis of third-party punishment: intervention on behalf of an affiliated conspecific in the presence in expectation of receiving support sometime in the future. Such an economy of justice, characterized by a long-term frame of reference, could serve as an adaptively viable basis of interaction in many animal (and human) societies.

### Dominance as a moderator of punishing behavior

Finally, we should mention that one characteristic of punishment in animals hints to a solution to a problem that arises in understanding the mechanisms of cooperation in humans. Punishment has costs in terms of time and energy, and if the benefit of punishment does not outweigh its expenses, it will not be adaptive. It is no wonder, then, that animal punishers are, for the most part, the dominant members of the group, which are the main beneficiaries of the cooperation of the punished (Clutton-Brock and Parker, 1995). They control the resources available to the group, have the right of way in accessing food reserves and hunting grounds, and are at the forefront of the breeding queue (sometimes exclusively). Therefore, it is adaptive for them to invest in punishment. Below we will consider the implications of these dynamics for models of punishment systems in human societies.

### Alternative perspectives on animal punishment

As noted, some researchers have questioned whether behaviors such as those described above should be called “punishment.” An alternative interpretation calls them “harassment,” since in many cases the actions result in immediate changes in the punished animal’s behavior, but there is no clear evidence that the punishment changes the punished animal’s behavior in the long run (Stevens et al., 2005). This terminological debate has of course revolved around the intuitive definition of “punishment,” and it is possible that instead of determining the optimal use of this term in animals based on models from the human world, one should act in the opposite direction. Riehl and Frederickson (2016) have argued that several of the above-cited cases are better seen not as punishment for cheating, but as protection of resources for the benefit of the “punisher,” and further argue that punishment evolved as a precondition for stable cooperation rather than as an evolved response to cheating.

Keeping those caveats in mind, the punitive behaviors of other animals can thus be summarized as follows: they are mainly expressed in relationships between members of the same species, especially among social animals; they help maintain social order; they are often conducted by the strongest members of the group; they address “crimes” that parallel human acts of theft, resource exploitation, and territorial invasion; they can be altruistic. Most importantly, these behaviors appear to be innate, and are expressed in particular situations that yield the relevant behavior. Although we have no clear knowledge of the level of awareness of animals and their character, it is difficult to assume that monkeys, crows, and bees alike act out of conscious insight when punishing other members of their species. However, such behaviors make great evolutionary sense, because they are adaptive, i.e., they contribute to survival and reproduction.

## An evolutionary approach to understanding punishment in humans

Punitive phenomena observed among animals present us with an important question: what underlies the punitive behavior of humans? This question is relevant both to the reactions of an individual in interaction with another person and to a person acting on behalf of a social group: legislator, judge, or any other person with punitive authority – teachers, military commanders, and even parents adjudicating conflict between their children. Are our intuitions about punishment – that we act out of values and a sense of justice – true, or are the motives rooted in instincts similar to those of other animals? After all, humans were shaped by the same evolutionary forces that created the other species, and our survival and reproductive success are as important to us as to any other animal. Are the reasoned explanations we give for our decisions in the area of punishment primarily post-facto justifications of innate tendencies? To address this question, I will present some contemporary findings about the punitive behavior of humans under controlled conditions, and then describe work regarding its biological infrastructure. Studies dealing with the question of the biological elements of punishment in human interactions have recently been extensively reviewed (e.g., Seymour et al., 2007; Du and Chang, 2015; Wu et al., 2022), so I will confine myself to noting some important trends that have been described in the literature.

### Punishing exploitative agents

Under controlled laboratory conditions, humans have been found to exhibit a strong tendency to punish others who do not cooperate for the common good, even if such punishment carries a cost (monetary, in most studies), and supposedly even when such punishment is altruistic (it does not benefit the punisher). Relevant evidence comes from the field of economic games, in which participants are assumed to behave in a way that brings them the most benefit at the lowest cost. Seemingly, deviations from optimal behavior in such games can imply rational failures, or other explicit values and implicit processes that drive participants’ choices beyond the immediate reward they receive.

In public goods games, there is a strong tendency to punish exploitative players who do not contribute their share for the benefit of the group (Fehr and Gächter, 2000). Players punish those who did not cooperate equally after each round of play even when it involves a cost to themselves (de Quervain et al., 2004). Some studies have shown that punitive tools not only lead to greater cooperation, but despite its cost, also improve the average profit of all players across many rounds of interaction (Gächter et al., 2008). This finding is important, as it confirms the claim that complex punitive behavior (i.e., behavior that also includes second-order punishment and so on) is adaptive and improves the viability of the society in which it is practiced, even when it carries a cost to the punisher. Moreover, mathematical models show that especially in cases when individuals have the option of joining or avoiding social action altogether, there is a rapid adaptive advantage to punitive behavior (compared to passive cooperation without punishing exploiters), even if it comes with a cost (Hauert et al., 2007).

Other studies show that the tendency to punish non-cooperating free-riders occurs even in conditions where the punisher not only pays a cost but will not benefit from “correcting” the exploiting person’s behavior. This phenomenon was demonstrated in a study in which people played several rounds of a public goods game, but with different players in each round, and without participants receiving information about the behavior of the players in previous rounds. Although under these conditions the costly punishment could not increase the chances of the punishers benefiting from improved cooperation of the punished players in the next round of the game, the players continued to punish and bear the cost involved – a case of altruistic punishment (Fehr and Gächter, 2002). Such punitive behavior observed under controlled laboratory conditions, might be attributed to the “sense of justice” present under ecological conditions, including feelings of anger and moral outrage (Wu et al., 2022), which also affects willingness to engage in altruistic third-party punishment (Ginther et al., 2022). Alternatively, costly punitive behavior has been attributed to reputation benefits of altruistic punishers (Mifune et al., 2020; Redhead et al., 2021). It has been noted, though, that while individuals can gain a great deal of reputational benefit from engaging in third party punishment, these benefits are only open to dominant individuals (Gordon et al., 2014; Gordon and Lea, 2016).

These studies demonstrate the effectiveness of punishment as a tool for optimizing social cooperation even in fairly artificial settings. The laboratory models involve the main characteristics of life in the public domain: competition versus cooperation, activity for the common good done voluntarily or out of necessity, and careful monitoring of differences in success within a group working to improve the economic situation of its members. Most of all, we see that punitive behavior is guided by cost-benefit considerations, in accordance with the utilitarian approach.

It should be noted that some doubt has been expressed regarding the above findings (e.g., Raihani and Bshary, 2019), as the cases in laboratory studies in which third-parties have been observed to engage in costly punishment might include conditions or demand characteristics that yield such behaviors when they would not be expressed under ecological conditions. These include: assigned roles and task-structured expectations, the presence of observers which introduces reputational considerations, and difficulty in distinguishing between responses motivated by (righteous) anger and those motivated by envy (Pedersen et al., 2013). Furthermore, in other studies in which vengeful motives or demand characteristics were eliminated, participants showed little or no altruistic costly punishment (Kurzban et al., 2007; Carpenter and Matthews, 2013; Pedersen et al., 2018).

Furthermore, there are many complexities in peer punishment situations in the absence of ancillary social institutions that can impact on the effectiveness of punishment in optimizing benefits for participants. Punishment can only be effective in the long run if it is cheap for the punisher and costly for the punished [as in studies reviewed by Chaudhuri (2011)]. In a fair game, responsibility for punishment should be evenly distributed among the players, and this can be ensured by second-order punishment (punishing those who do not punish first-order exploiters, and so on). However, the spiraling costs of infinite-order punishment can make it prohibitively expensive and vitiate its advantages. Moreover, the possibility of retaliation significantly reduces willingness to punish (Nikiforakis and Engelmann, 2011). Indeed, it has been noted that direct punishment of non-cooperation is rarely observed outside the laboratory [e.g., studies cited by Gordon and Puurtinen (2021)]. These complications may explain while so many societies have developed centralized institutions, based on pool contributions, for the administration of punishment; more on this below.

Even if absolutely altruistic costly human punishment is rarer than claimed by its advocates, under ecological conditions of an interactive society, criminal behavior can always be perceived as potentially damaging to the welfare of every individual, and maintenance of the social order is requisite for the survival and thriving of every member of society. Thus, even when a person is not the direct victim of a criminal act, witnessing or becoming aware of such crimes might recruit inherent tendencies to punish offenders. In an animal model of such dynamics mentioned above (cleaner wrasses), individuals punished conspecifics whose exploitation of symbiotic animals terminated the symbiotic opportunity for the punisher (Raihani et al., 2010). Accordingly, to the extent that a criminal hurts a victim in such a way that indirect harm is caused to the rest of society (e.g., because of social commitment to the rehabilitation of the victim), we may act to punish that criminal because of a human analog of such motivation.

### Preventing resource control by dominant individuals

A different evolutionary approach to understanding human punishment behavior comes from attempts to explain the difference between the high reproductive fitness status of physically dominant males in non-human primate societies and absence of that situation in humans (Gintis et al., 2015; Wrangham, 2018). It is suggested that in human societies, coalitions of non-dominant males began to join together to intimidate, expel, or even kill domineering individuals who attempted to monopolize reproductive opportunities. This “counter dominance hierarchy” (Erdal and Whiten, 1994) is asserted to be universal in nomadic hunter-gatherer societies that ostensibly provide a glimpse into human group dynamics preceding the agricultural revolution and the higher-density civil societies which it engendered. Starting from this point of departure, Boehm argues that capital punishment – in the sense of communally approved killings of group members – developed in the Pleistocene era, enabled by shared intentionality (perhaps afforded by the development of language; Gavrilets et al., 2008) and the technological advance in force-multiplying weapons. Indeed, in traditional nomadic hunter-gatherer societies, the most likely targets of capital punishment are antisocial males with a history of selfish aggression (Boehm, 2012). Such capital punishment is asserted to be a cultural universal (Otterbein, 1986). Moreover, the evolution of capital punishment (and other cooperatively-imposed punishments) can contribute to explaining why humans exhibit comparatively low tendencies toward reactive aggression (Wrangham, 2018). To the extent that human individuals have been “off-loading” responsibility for punishing exploitive conspecifics to the group, each individual’s need to engage in costly punishment declines. Boehm (2012) attributes this to an incidental genetic consequence; I would argue that the group selection pressure for prosocial norms and parochial altruism (Gintis et al., 2015: Henrich, 2016) can work more effectively when the institution of collaborative punishment liberates individuals from engaging in reactive aggression to protect reproductive and other resources, and serves as an anchor for other collaboration. This view seems to be supported by the study of Molleman et al. (2019), who report that people’s decisions to punish in a public goods game are strongly contingent on other participants’ punishment decisions.

### Leveling

Yet another explanation of the seeming tendency toward altruistic punishment is that it is intended to optimize the status of the punisher in current circumstances of competition with the offender, rather than to ensure future cooperation (Raihani and Bshary, 2019). In this approach, punishment has a payoff-leveling goal, such that third-party punishers can rationally invest resources in punishing in order to reduce fitness differentials between themselves and bad actors who have improved their relative resources standing by exploiting others. Adduced in support of this approach are findings that punishers are not more likely to be cooperative than non-punishers (e.g., Weber et al., 2018); that punishment does not seem to reliably convert defectors into co-operators (e.g., Barclay and Raihani, 2016); and that third-party punishers seem to respond more to the results of the defection for the welfare of the defector rather than the loss incurred by the victim (Raihani and McAuliffe, 2012).

### Exclusion and ostracism

One punishment strategy that achieves many of the societal benefits of prosocial punishment is social exclusion (Gruter and Masters, 1986; Sasaki and Uchida, 2013). Since humans evolved to thrive under conditions of social cooperation, depriving offenders of the opportunity to engage with others is an effective means of punishment. It has been demonstrated that in certain formats such public goods games, exclusion can be a more powerful strategy for optimizing social cooperation than active punishment (Liu et al., 2017). Historically, ostracism or banishment has been used to punish offenders of a range of social norms (Williams, 2001). The threat of exclusion from the intimacy of a family circle, or loss of reputation in a limited social group, may serve as a strong deterrent for many offenders and offenses, and are likely to satisfy the desire for revenge or expression of revulsion at betrayal of trust by an offender who is part of such a family or closed social group. More generally, organized group ostracism – the ecological analog of exclusion in economic games (Sasaki and Uchida, 2013) – can be successful in deterring major offenses committed against other members of society. For example, cheating in trading activities might be punished by exclusion from engaging in such economic activity temporarily or permanently. More radically, organized ostracism might take the form of deportation (though finding another state or country willing to take in the offender is much rarer in the modern information-available world than it had been in earlier times).

### Punishment by dominant members of society

As noted above, dominant members of animal societies are most likely to be the agents of punishment, as in doing so they conserve resources to which they have preferential access. In human history, dispensing justice has traditionally been the responsibility of the ruler. The chronologically earliest attestations of this notion are from civilizations which left extensive written records. We find the idea in the claim by Sargon of Akkad, who establish the world’s first empire in Mesopotamia *circa* 4,300 years before present, to having served as a dispenser of justice. This trend is also attested by surviving legal codes from rulers of Mesopotamia such as Lipit-Ishtar and Hammurabi (3,600 years ago; Court, 1995). Importantly, the ancient monuments from which we know about these law codes represent the rulers as receiving them from the gods – implying that they represent an objective system of justice rather than the whim of the ruler. In ancient Egypt, Pharoah was held to be both divine and ultimately responsible for justice (Frankfort, 1978)^7^.

As far as other cultures that did not leave verbal records, we cannot know the full extent of ruler-justice relationships. Contemporary and more recent hunter-gatherer societies are often examined as a source for how pre-modern societies functioned in general (Garfield and Hagen, 2020). The general trend of findings is that in many small-scale societies, tribal or band leaders are commonly responsible for resolving within-group conflicts (Glowacki and von Rueden, 2015). In Tsimane agricultural society, members of society who enjoyed the most political power adjudicated the majority of social conflicts among members of their group (von Rueden et al., 2012). Such power-punisher role connections have been documented for other North American first peoples such as the Kapuku and the Cheyenne (Singh and Boomsma, 2015). Such dominance can be achieved by election and be temporary: when Arapaho bands came together for the annual summer buffalo hunt, they elected a tribal chief to oversee the animal harvesting and to police crime; that was the only time of year in which there was a leadership figure with that level of sanctioned authority (Lowie, 1948).

Having noted these historical trends, it has been proposed that in “ancestral” conditions, benefits to reputation and maintenance of cooperative relationships make widely practiced cheap punishment a generally beneficial behavior (Scott-Phillips et al., 2011). As social groups grew, increasing both the overall resource pool and the number of participants in the dynamics of its distribution, punishing becomes more expensive and comes to be transferred to leadership figures (Hooper et al., 2010). Ultimately, decoupling the authority to punish societal offenders from power advantages accruing personal benefit, leading to egalitarian judicial and enforcement institutions characterizing some modern societies, is arguably an incremental development in the trend toward effective societal equity. Below I will argue that maintaining this social contract is vital to the coherence of contemporary social institutions.

## Brain infrastructures of punitive behavior in humans

In evolutionary biological thinking, it is customary to distinguish between proximate causes and ultimate causes. Maximizing survival and reproduction is the ultimate cause of the behavior of an entire species of animal, but any ultimate cause must be served by biological mechanisms characterizing individuals of that species, which are the proximate causes of any given behavior. In our case, the adaptivity of punitive behavior is an ultimate cause, which explains why it developed and is maintained by evolutionary processes. However, it remains to find the immediate cause of the punitive behavior in the person acting in any given situation. What motivates people to punish others is not the ability to perform statistical calculations that prove the effectiveness of punishment in the long run, but an intuition, which they sometimes call a “sense of justice” or “sense of fairness.” How does the evolutionary urge to punish translate into a mechanism that motivates people to choose to punish offenders?

### Reward motivation for punishment

One possible mechanism serving as a proximate cause for punitive behavior was identified in a study in which participants played a computerized version of the public goods game while their brain activity was mapped using PET. In cases that the players planned to impose effective punishment on exploitative players, activity was recorded in the striatum, an area important for learning the reward value of actions. Furthermore, participants who exhibited stronger activity in the reward-related area to were willing to pay a higher personal cost to punish free-riders (de Quervain et al., 2004). The sense of reward is therefore a proximate cause that gives an incentive to punishers even without complex calculations of long-term cost and benefit (Seymour et al., 2007).

Another study dealt with the brain mechanisms involved in assessing the fairness of the behavior of others and the response that results from this assessment (Sanfey et al., 2003). This study used a two-player ultimatum game, in which the proposer receives a sum of money, and offers part of it to the responder. The responder can accept the part offered to him, and then both players divide the sum accordingly, or reject it, and then neither player gets anything. According to the rules of economic logic, especially if only one round of the game is played, the responder should agree to any offer offered to him, since any amount they receive will be greater than zero. In practice, however, meager proposals are usually rejected. There is an interesting cultural variance in what is considered fair (Henrich et al., 2006). Studies conducted in Western countries found that proposals of less than 20% of the amount were rejected in half of the cases, but in other cultures proposals were considered fair and accepted only if they gave the recipient of the offer an even higher percentage. Rejection of an insufficient offer can be seen as a type of costly punishment, since the decision-maker prevents the bidder from receiving the reward by waiving his own reward (Roth, 1995).

Sanfey et al. (2003) reported that unfair offers in this game were accompanied by heightened activity in several brain regions: anterior cingulate cortex, anterior insula, and dorsolateral prefrontal cortex. They suggested that activity in the anterior insula was responsible for the decision to reject unfair offers. The anterior insula is associated with negative emotional states – pain and distress, hunger and thirst – as well as arousal of the autonomic system. The insula has been documented as also being involved in stimulus valuation and representation of feelings of anger and disgust (Craig, 2008). Accordingly, as participants’ insular response marked the offer received as unfair, the tendency to punish the proposers by denying them any profit from increased. This finding converges on others indicating the important role of emotional processes, and their neural infrastructures, in fairness judgments in the context of economic interactions and in shaping our responses to unfair behaviors (Seymour et al., 2007). It should be noted, though, that a subsequent meta-analysis did not confirm the primary role of the insula in rejecting unfair offers, instead highlighting a number of areas that were identified as implicated in social cognition (Gabay et al., 2014).

These studies give us an indication of the brain mechanisms leading to retaliatory punishment behaviors by recipients of unfair treatment. Above and beyond such responses, several studies have further begun to identify the processes and neural substrates of costly third-party (altruistic) punishment. Strobel et al. (2011) report that activity in reward-related brain areas such as the nucleus accumbens is correlated with third-party punishment in the context of the Dictator Game. Other regions implicated include dorsolateral prefrontal cortex, anterior cingulate cortex, insula, and caudate nucleus. Furthermore, they found that some of these activation effects these were sensitive to dopaminergic function due to genetic Val158Met genotype variation in the COMT gene. Similar brain areas were implicated in other studies (e.g., Feng et al., 2015; Stallen et al., 2018). Corradi-Dell’Acqua et al. (2013) reported that while the medial prefrontal region was only associated with vengeful punishment, the anterior insula activation was associated with fairness-related third-party punishment as well. Hu et al. (2015) report that ventromedial prefrontal cortex was also implicated in third-party punishment, but add the observation that both third-party helping and third-party punishing activated the reward-related striatum bilaterally, suggesting that these altruistic actions share a common neuronal basis.

### Empathic modulation of third-party punishment

One brain substrate of altruistically punitive behavior may be related to the putative mirror neuron system (Rizzolatti and Craighero, 2004). Several researchers claim that mirror neurons are an important basis of empathy. Mirror neurons in brain areas associated with an emotional experience can cause us to feel feelings that we would feel during a particular experience even when we are watching another person go through the same experience (Rizzolatti and Craighero, 2005). Based on the finding of Strobel et al. (2011) of insula activity related to third-party punishment, it is possible that empathy with victims based on insular mirror-type neurons leads us to such execute altruistic punitive actions (Damasio, 2003).

Empathic considerations may affect not only our attitude toward the victim but also our attitude toward the offender in cases where we may identify with them. This may explain why though in principle people believe in harsh deterrent penalties for hard-to-detect offenses, they still mitigate penalties when given sympathetic information about the offender’s motives, e.g., that they embezzled corporate funds to pay for emergency surgery for their nephew (Carlsmith et al., 2002). Relatedly, decisions to refrain from punishing offenders in the Justice Game was associated with activation in the temporo-parietal junction, an area associated with perspective-taking. In that study, punishment severity was modulated by the administration of oxytocin, a neurohormone related to affiliative behaviors, possibly by shifting punisher focus from self-interest to group norms (Stallen et al., 2018).

The empathic modulation of third-party punishment is demonstrated by the effect of heightening feelings of compassion toward people who are suffering on increasing punishment likelihood and degree (Pfattheicher et al., 2019). Similarly, people who underwent an intervention to induce an emotional state of gratitude were more likely to engage in costly punishment of an unfair player in a Dictator game than those in whom feelings of happiness were induced, or in a neutral condition (Vayness et al., 2020). It may be argued that such findings support an understanding of third-party punishment as concern regarding the victim and not just vengefulness toward the perpetrator.

### Emotional prediction error

Another modulator of third-party punishment which is intimately related to neural mechanisms is prediction error. Heffner et al. (2021) present evidence that emotional prediction errors caused by unexpectedly negative valence and unexpectedly strong arousal independently increase costly punishment in Ultimatum and Justice Games. As the third-party in these studies was acting directly on behalf of a theoretical responder, it might be that the emotional dynamics leading to punishment were not completely altruistic, but rather an insult to the participant’s sense of agency. It remains to be seen whether such prediction errors will be found to affect thoroughly altruistic costly punishment.

A further word of caution regarding claims based on brain imaging, psycho-pharmacological, and genetic findings is required, as in many studies, costly forms of punishment in one-shot interactions, even when the punisher is a direct participant are often called altruistic punishment (e.g., Gabay et al., 2018; Mussel et al., 2018). It seems more appropriate to reserve the label ‘altruistic’ for third-party costly punishment only, reserving the term ‘spiteful punishment’ for costly rejection of unfair offers (Yamagishi et al., 2017).

We have thus seen that in humans, too, a proximate cause can be found for punitive behavior: brain mechanisms that begin with emotional reactivity to actions that harm oneself or another, go on to mobilize systems of aggression to promote reactive response, and end with a feeling of satisfaction that justice has been served. Expectation of the rewarding feelings that follow punishment is the primary and basic motive for the punitive behaviors, even before the person formulates a clear conceptual analysis regarding the case in question and the ultimate practical value of the punitive action.

## Punishment in interpersonal interactions vs. punishment by a society’s legal system

These findings lead us to the main argument I would like to make in this article: that above and beyond the consequentialist and retributive factors mentioned above, there is another consideration that must be considered when determining penalties for offenses. Punishing offenders is intrinsically rewarding, and when people (and other animals) do not get the reward they expect to receive, it is biologically equivalent to a punishment (Seymour et al., 2007; Sigmund, 2007). One can understand the negative value experienced in terms of a citizen’s frustration in thinking of a criminal who has not been caught or punished. Therefore, my argument is that just punishment has a positive, utilitarian value for the whole of society – for citizens who are aware of punishment for offenses – even beyond the ability of punishments to prevent future offenses.

### Punishment and social cohesion

Among all the considerations that must be taken into account when determining penal policy in general and when applying it in specific cases, one must also consider the individual’s sense of reward from the just punishment. This is not an exercise in giving cheap satisfaction to instinctual impulses, but another element in the well-known argument that citizens will cooperate more closely with the system of government and justice if they feel that it succeeds in enforcing the law as equally and fully as possible (Tyler, 2003). Identifying the biological processes of receiving a reward by performing punishment helps us understand the biological glue that holds the system together. If members of a human society delegate the power of punishment to a public system, and this system does its job faithfully and efficiently, the citizens of the society gain the sense of reward that they would receive in other circumstances by performing the necessary punishment on their own^8^. Furthermore, effective punishment of offenders prevents citizens from the frustration that causes a negative feeling, which can certainly affect their participation in social participation.

One suggestion regarding the basis for the willingness of members of society to engage in costly third-party punishment is that such behaviors provide a costly signal of trustworthiness (Jordan et al., 2016). This possible basis of the origins of costly punishment may have implications for societal institutions. To the extent that a government and its agencies is perceived as using whatever resources are necessary in order to punish exploitative behaviors, that might increase citizens’ perception of governmental trustworthiness and the willingness to cooperate with it. Indeed, the institutionalization of punishment engenders a reduction in the prevalence of individual acts of revenge (Duntley and Shackelford, 2008; Jackson et al., 2019) and the possibility of the transformation of cultures of revenge (sometime called “cultures of honor” (Nisbett and Cohen, 1996) into cultures of societal protection of individuals.

### Social punishment relieves members of society of negative emotions

By shifting the responsibility for punishment from the individual to the institutions of society, when we believe that the system is effective in apprehending and dealing with miscreants, we liberate ourselves from obsessively thinking about crimes committed (which otherwise is an adaptive process motivating us to take action against those who appropriate our resources). If we understand that human beings have a basic tendency to punish, to the point of being ready to execute altruistic punishment, positive psychology can give us another important insight. Transferring responsibility for punishment to social institutions allows us to begin processes of forgiveness of an offender (as expressed in statements such as: “The criminal has paid their debt to society.”). Such a process of forgiveness is not only beneficial to the miscreant, but emotionally healthy for us as well (Seligman and Csikszentmihalyi, 2000). As noted by Jackson et al. (2019): “Despite the short burst in positive affect immediately after a vengeful act, it only takes a few minutes for avengers to begin reporting regret, rumination, and negativity.” Such moderation of individual reactivity to criminal acts may be related to the findings that the presence of others decreased the severity altruistic punishment decisions, ostensibly due to diffusion of responsibility, and that this diminution of severity was accompanied by altered neural responses in dorsal anterior cingulate cortex and modulation of other brain responses (Wang et al., 2017).

The transfer of responsibility for punishment from the individual to the institutions of society enables the members of society who feel threatened by criminal behavior and intuitively respond with a desire for revenge an added benefit. When revenge is carried out by an individual, it may be motivated by anticipated feelings of satisfaction for redressing a wrong done to them (Chester and DeWall, 2017). However, this pleasant affect is often quickly replaced by negative affect, possibly because of the fear of reciprocal retaliation (Akın and Akın, 2016). When personal satisfaction in redressing wrongs is mediated by powerful joint institutions, the latter consideration is less relevant.

### Implications for types of punishment

The approach proposed here has important implications for the types and degree of punishment. Consideration of the biological basis of punishment leads us to consider the price that we – the society of punishers – are willing to pay in our attempt to prevent anti-social actions. The notion of a punishment fitting the crime might be applied to such considerations – not in the conventional sense of what is appropriate for the criminal, but in this biologically-informed sense of what serves the needs of the general public. We should not be interested in the offender getting what they deserve; the key consideration that should interest us is the effectiveness of the punishment in obtaining the common good – whether as a deterrent, as preventative immobilization, or in providing biologically-grounded satisfaction for members of society – all according to utilitarian considerations.

Another consideration that should affect the type and amount of punishment is the cost of punishment relative to the benefit inherent in it in terms of deterrence, prevention or compensation. The cost of lengthy prison terms should be weighed against the benefit gained from them. The biological-utilitarian approach can perhaps lead to a more creative expansion of the pool of punishment methods – for example, in the direction of compensating victims directly by the criminal who harms them, or using means that are currently considered inappropriate in many societies, such as causing pain or physical harm. The emphasis should be not on the offender, but on the good of all members of society. Caution is required: the dangers inherent in the actual use of physical punishment are very clear, and require careful reviews to ensure the safety of the innocent. But even the use of imprisonment has risks, and the loss of individual freedom occurring in decades-long prison sentences can be considered a more serious violation than corporal punishment (and the interpersonal differences in this matter can be very large).

### The public arena of punishment

Another implication of the biological-utilitarian approach to punishment concerns the nature of the arena in which the punishment is carried out (Stolzenberg and D’Alessio, 2003). In the twenty-first century, no doubt many will object to flogging criminals in the town square. But punishments that take place away from the eyes of society may not achieve the much-needed benefit of deterring potential criminals. Moreover, when punishment is not public, ordinary citizens do not get a sense that criminals are being punished for their actions, which leaves members of society with the sense of frustration. Consideration should therefore be given to how public exposure to punitive performance can be obtained with respect to any type of punishment that society decides to use. Admittedly, some criminal trials receive extensive media coverage, especially when it comes to particularly heinous crimes or the offenses of famous people. But for the most part the criminal justice system and the punishment branch operate outside the spotlight of public awareness. Some people may believe that this is better, but the consequences of such an approach must be taken into account. A meta-analysis of studies examining the effectiveness of deterrence in punishment found that while publicity-rich punishment does not minimize homicides, it has deterrent value in cases of property crimes, traffic offenses, and administrative violations (Dölling et al., 2009).

A fascinating demonstration of the very basic nature of the desire to witness justice being done is provided by the study of Mendes et al. (2018). They enabled chimpanzees and 4-, 5-, and 6-year-old children to learn through interactions that a certain agent was either pro-social or anti-social. Subsequently, they had the opportunity to witness those agents being punished, but at a cost (tokens for children and physical effort for chimps). Chimps and 6-year-olds were more willing to engage in costly viewing of the punishment of anti-social agents. Seemingly, the need to see offenders receiving their just deserts is ontogenetically and developmentally very basic.

### Preventing retaliation

The transfer of responsibility for punishment from each participant in a society to the societal institutions also addresses the problem of the counter-reaction of the punished. A mathematical model shows that in the development of a group, when punished persons can respond and punish the punishers, the cost of the primary punishment causes punishers to cease their activity and leads to the disruption of cooperation within the group (Janssen and Bushman, 2008). There are various solutions to this problem, such as anonymous punishment that does not allow for a targeted counter-reaction. In traditional societies the punishment is usually done by a leader who is generally the most powerful in the group and therefore less vulnerable, or by a leader with traditional powers who enjoys the support of the rest of the group. In both cases, due to their status the leader benefits from an expanded share of the group’s resources. They may therefore invest resources in punishment because they will get a larger share of societal resources that are not selfishly utilized by free-riders who do not take part in investing resources or working for the group. In modern societies, law enforcement agencies are usually given special protection against punitive responses (as evidenced by the many resources invested in apprehending criminals who have harmed police officers and judges and the over-punishment provided for them by law), and also receive special law enforcement resources. In these ways, the institutions of modern society have inherited the roles and resources of traditional leadership in small societies. Seemingly, such a Hobbesian “Leviathan” is necessary in all stable large-scale societies.

### Administering punitive institutions

Another aspect of the institutionalization of punishment is the judicial structure used to assess culpability and the nature and degree of sanction to be applied to an offender – e.g., jury vs. judge trials, numbers of judges, separation of verdict and sentencing. There is wide range of variation across societies in these parameters, and the question of which choice is operationally optimal is a complex one. One factor which should be added to the range of considerations is that in many studies of peer punishment, punishment decisions that are made by consensus of participants are more effective in engendering subsequent cooperation (Eriksson et al., 2017; Raihani and Bshary, 2019). This might suggest that jury systems might be more effective in achieving the ultimate goals of punishing, even if they are less effective in applying the principles of law or in assessing evidence. Additionally, there is ethnographic evidence that cooperation following upon sanctions imposed by elected punishers was greater than when the monitoring role was assigned by the organizers of the situation (Baldassarri and Grossman, 2011). This might be an argument for the importance of judicial elections at all levels, to be considered alongside the problems of partisan and opportunistic politics in judicial appointments.

Of course, giving some members of society authority to judge and punish others may result in those persons abusing their power and privilege for their own benefit – in other words, receiving bribery. When violators can choose to bribe corrupt enforcers to avoid being punished, the societal institutions may decay rapidly (Muthukrishna et al., 2017; Liu et al., 2019). Indeed, the potential for corruption among those in power might be responsible for the limitations demanded on punishment severity (Abdallah et al., 2014). Unsurprisingly, the effectiveness of any judicial system seems to require full transparency and constant vigilance of the entire society (Muthukrishna et al., 2017; Lee et al., 2019).

### Idealization of motivations for punishment

As I noted above, Carlsmith et al. (2002) show that ordinary citizens often state that what guides them in thinking about punishment are utilitarian considerations (such as prevention), not considerations of doing justice. But when given cases to judge whose decision depends on the preference for one of the above considerations, they usually act on considerations of doing justice (or perceived appropriateness of punishment), rather than on considerations of prevention. A brain systems approach can help account for this discrepancy. Rational thinking of the type that traditional legal theory deems appropriate for judgment is conventionally ascribed to prefrontal circuits, especially the dorsolateral prefrontal region, which Buckholtz et al. (2015) assign the role of integration of information about culpability and harm. However, in considering a particular case with all its human factors, we recruit imagination, emotion, empathy, and autonomic responses which color our thinking. Each of the systems supporting those aspects of our mental lives can be differentially recruited depending on the elements of the case under consideration. Insight into how separate brain systems for different components of punitive thinking may be identified came from a study that tracked the brain activity of people who were asked to judge offenders for crimes that differed in the severity and degree of responsibility of the law breaker (Buckholtz et al., 2008). Activity in amygdala, medial prefrontal cortex and posterior cingulate cortex was found to correlate with severity of the penalties imposed on offenders. In contrast, dorsolateral prefrontal cortex activity was more closely related to ascription of the degree responsibility involved in the offense. Another study reports the involvement of dorsolateral prefrontal cortex when sentencing decisions are made in a state of direct personal interaction (Knoch et al., 2006). These findings support the claim that there are common mechanisms that influence sentencing decisions when we are personally involved in a case and when we act as a third party.

Emotional representations of value influence decision-making in many areas of life, acting automatically to enable us to response adaptively (Damasio, 2003). Judges, jurists, and enforcement officials are likely to be influenced by such types of decision-making processes when they are dealing professionally with crime, even when they are not direct parties. The transfer of responsibility for punishment to the institutions of society does not prevent the individuals responsible for doing justice from punishing according to their instinctive responses, which are guided by emotions generated by the same brain processes that have served us and our predecessors across evolutionary time. Judicial deviations from the “dry” provisions of a legal code, which are explained as the exercise of the judge’s or authority’s discretion, may in fact be based on intuitions based on emotional biological systems, and not necessarily on purely logical considerations. Awareness of these effects can enhance the judicial process.

## Summary and conclusion

In this article I have cited some evidence from ethology and brain research which provide insights into the complex mechanisms responsible for our thoughts and behavior in the area of punishment. My main contention is that the need to punish criminals is embedded in our biological nature, that the satisfaction of this need is a social need, and the punishment policy should be influenced by this, alongside other considerations. Ignoring such considerations may lead to societal frustration that can undermine support for societal institutions, with the concomitant decline in compliance and cooperation. It is sometimes argued that evolutionary and neurobiological accounts of punishment behaviors undermine retributivist approaches to punishment (Wiegman, 2020). I would argue that this is not necessarily so: since punishment is a function of human social nature, people’s retributive intuitions – whatever their metaphysical status – are just as biologically important as consequentialist considerations.

One might argue that this approach panders to the baser aspects of human nature, and that we should reject the Old Testament notion of ‘a life for a life’ in favor of the more pacifist New Testament approach attributed to Jesus of Nazareth, “You heard that it was said, ‘An eye for an eye and a tooth for a tooth,’ but I say to you, do not resist the one who is evil. But if anyone slaps you on the right cheek, turn to him the other also.” (Matthew 5:38–42). But as I have mentioned at the outset, punishment is not a value in its own right, and in most cases its use means that a deeper failure of societal institutions has occurred. It is hard to accept the harsh realities of effective punishment, but until we build truly just societies, we cannot dispense with “dispensing justice.” Understanding the evolutionary and neural underpinnings of the relevant processes might not lead us to champion different values, but might instruct us in how to better translate them into practical strides for maintaining public order. As Greene and Cohen (2004) wrote about the impact of brain research insights on understanding free will and about the implications this has for the justice system, understanding the biological basis of punishment doesn’t change anything, but it changes everything.

## Author contributions

DL did the research and wrote the manuscript. The author confirms being the sole contributor of this work and has approved it for publication.

## Footnotes

1 In the following explication of these approaches, I have drawn some opinions and commentary from Israeli legal precedents and supporting literature. The Israeli legal system is based on Anglo-American Common Law foundations, but includes aspects of Ottoman Turkish law (as the territory of Israel was formerly under Ottoman rule), and of Jewish jurisprudence as expressed in Talmudic and Rabbinic literature. It is therefore a source of historically and culturally complex legal notions, instructive for illustrating the ideas in question. This is also an opportunity to give legal scholars some exposure to this otherwise less-known literature. I hope it will be instructive to all readers.

2 And in the case of some sex offenders – through chemical or surgical castration.

3 In Israel, the Biblical adage “As he has done, so shall be done to him” (Leviticus 24:19) is often cited as a historical-cultural foundation for this approach.

4 Other factors, such as expressions of regret about the crime, are fundamentally important for the retributive approach, but can be appropriate considerations for the consequentialist approach as well.

5 Several of the cases described in the following sections are drawn from this seminal paper.

6 Examples of this can be found, *inter alia*, in the laws of Hammurabi (Babylon, seventeenth century BCE). According to one of the sections, if a trader holds a free man in prison against his will on the grounds that he owes him a financial debt, and the prisoner dies from beatings or abuse, the trader’s son must die in response (Section 116). Also, if a man strikes a free woman and she dies, she the daughter of the slayer is to be killed (Section 210). The Hebrew Bible tradition seems to refer to this principle in order to reject it. Regarding retributive justice following a person’s being gored to death by an ox, it is stressed that “… also if a son is gored or a daughter is gored, this sentence will be done [i.e., the owner of the ox, and not a member of their family, is liable to punishment] (Exodus 21:31).” Though some Biblical tradition explicitly rejects the Babylonian approach (“Sons shall not be put to death for [the offenses of] fathers, nor fathers for [the offenses of] sons: a man for his sin shall be put to death” (Deuteronomy 24:16). Elsewhere this approach is identified as a reflection of divine justice: “For I am the Lord your God, a jealous God, visiting the sins of fathers upon sons, and upon the third and fourth generation for those who hate Me” (Exodus 20:5).

7 This theme is also echoed in the Hebrew biblical statement: “A king doth establish justice in the land…” (Proverbs 29:3).

8 This phenomenon is reminiscent of the biblical saying: “In the good of the righteous shall the city exult, and in the elimination of the wicked there is joy” (Proverbs 11:10).
